# Secondhand Smoke Correlates with Elevated Neutrophil-Lymphocyte Ratio and Has a Synergistic Effect with Physical Inactivity on Increasing Susceptibility to Type 2 Diabetes Mellitus: A Community-Based Case Control Study

**DOI:** 10.3390/ijerph17165696

**Published:** 2020-08-06

**Authors:** Yohanes Andy Rias, Christopher James Gordon, Shu Fen Niu, Bayu Satria Wiratama, Ching Wen Chang, Hsiu Ting Tsai

**Affiliations:** 1 School of Nursing, College of Nursing, Taipei Medical University, Taipei 11031, Taiwan; yohanes.andi@iik.ac.id; 2 Faculty of Health and Medicine, College of Nursing, Institut Ilmu Kesehatan Bhakti Wiyata, Kediri 64114, Indonesia; 3 Susan Wakil School of Nursing and Midwifery, Faculty of Medicine and Health, The University of Sydney, Camperdown 2050, NSW, Australia; christopher.gordon@sydney.edu.au; 4 Post-Baccalaureate Program in Nursing, College of Nursing, Taipei Medical University, Taipei 11031, Taiwan; niu@tmu.edu.tw; 5 Department of Nursing, Shin Kong Wu Ho Su Memorial Hospital, Taipei 11031, Taiwan; 6 Department of Epidemiology and Biostatistics, Faculty of Medicine Public Health and Nursing, Universitas Gadjah Mada, Yogyakarta 55281, Indonesia; bayu.satria@ugm.ac.id; 7 Department of Obstetrics and Gynecology, Taipei Medical University Hospital, Taipei 11031, Taiwan; cwchang967@tmu.edu.tw

**Keywords:** neutrophil–lymphocyte ratio, physical inactivity, secondhand smoke, type 2 diabetes mellitus, white blood cells

## Abstract

Secondhand smoke (SHS) and physical inactivity are thought to be associated with type 2 diabetes mellitus (T2DM), but the synergistic effect of SHS with physical inactivity and their relationships with T2DM–associated inflammation biomarkers have not been estimated. We investigated the roles of SHS exposure and physical inactivity and their synergistic effect on T2DM risk and their relationships with T2DM associated inflammation biomarkers, neutrophil–lymphocyte ratio (NLR) and white blood cells (WBCs). A case–control study was conducted in total 588 participants (294 case T2DM and 294 healthy controls) from five community clinics in Indonesia. Participants completed a standardized questionnaire on demographic information, smoking status, physical activity habits and food consumption. WBCs and NLR levels were determined using an automated hematology analyzer. Adjusted odds ratios (AORs) and 95% confidence intervals (CIs) were analyzed using multiple logistic regression model. The synergistic effect was analyzed using additive interaction for logistic regression. Physical inactive people exposed to SHS exhibited a synergistically increased 7.78-fold risk of T2DM compared with people who were not exposed to SHS and who were physically active. SHS is significantly correlated with a high NLR, WBCs and has a synergistic effect with physical inactivity on increasing susceptibility to T2DM.

## 1. Introduction

Type 2 diabetes mellitus (T2DM), a chronic metabolic disorder characterized by hyperglycemia [1], has reached epidemic levels globally and has increased the risk of mortality [2]. According to a report by the International Diabetes Federation, approximately 592 million people worldwide will have diabetes mellitus by 2035 [3], and this number will increase to 642 million by 2040 [2]. In Indonesia, the prevalence of T2DM has increased from 8.5–10.3 million in the period from 2013–2017 and will increase further to 14.1 million by 2035, possibly affecting as many as 16.6 million by 2045 [3,4]. Therefore, identifying potential risk factors of T2DM is strongly recommended. These factors include demographic characteristic regarding the disease and lifestyle behaviors factors. Consequently, these factors may increase inflammatory markers and risk of developing T2DM [5].

Smoking behaviors, including active smoking and exposure to secondhand smoke (SHS), were reported to be prominent risk factors for glucose intolerance [6,7]. Few studies, however, have estimated the damage that active smoking has caused the development of T2DM in Indonesian [8,9]. Researchers in Indonesia found that active smokers had a significantly increased risk of prediabetes compared with nonsmokers [8]. However, another study found a nonsignificant relationship between active smoking and T2DM [9]. Notably, more than 30% of nonsmokers are reportedly exposed to SHS, which contributes to approximately 1% of total deaths and 0.7% of the disease burden worldwide [10]. In Indonesia, approximately 78% of people at home, 83% of those visiting restaurants, and 50% of workers in the workplace are exposed to SHS [11]. A prospective cohort study in the United States reported that nonsmokers with SHS exposure had a 1.35-fold higher risk of having glucose intolerance compared with those with no exposure [7]. However, no epidemiological research has investigated the association between SHS and T2DM in Indonesia. Thus, research on the threat of SHS and the susceptibility of T2DM in Indonesia should be undertaken.

A meta-analysis revealed that 20 metabolic equivalent of task (MET)-h/week of leisure-time physical activity reduces risk of T2DM by 15% [12]. Another meta-analysis concluded that an increase of 100 min of physical activity per week is associated with an average change in fasting glucose of −2.75 mg/dL and an average change of −0.14% of glycated hemoglobin [13]. Moreover, physical inactivity was associated with increased abdominal adiposity, reduced energy balance [14], and increased glycated hemoglobin and fasting glucose levels [13]. In Indonesia, 46.4% of the population engages in low levels of physical activity [15]. Moderate physical activity level was found to decrease 10-year diabetes incidence by 53% compared to low levels of physical activity [16]. Furthermore, physical activity of 5.1–10.0 MET h/week revealed in significantly reduced fasting glucose, glycosylated hemoglobin and body weight as well as associated with a significantly lower risk of T2DM as compared with physical inactivity [17]. The etiological factors including higher consumption of carbohydrates and fat, low energy intake, and obesity, as well as physical inactivity associated with higher level of inflammation biomarkers including NLR and WBCs [18]. Moreover, physical activity of <7.5 MET-hr/week is a potentially vital factor for increasing high NLR and decreasing the quality of life in patients with T2DM. These conditions can trigger out of control hyperglycemia and contribute to deteriorating the process of T2DM [19]. We hypothesize that inactivity and/or a low volume of physical activity could be predominant risk factors for developing T2DM among Indonesians. More importantly, SHS and physical activity of <7.5 MET-h/week could synergistically increase susceptibility to T2DM. However, only one Indonesian study determined a 1.098-fold increased risk of prediabetes in physically inactive individuals compared with those with high or moderate levels of physical activity [8], and no study has examined the synergistic effect between SHS exposure and physical inactivity on the risk of T2DM. Hence, estimating the relationship between physical inactivity and T2DM as well as its synergistic effect with SHS of increasing the risk of T2DM in Indonesian was important.

Inflammatory abnormalities can be detected by an increase in white blood cells (WBCs) consisting of several subtypes, including monocytes, lymphocytes, and granulocytes (neutrophils, eosinophils, and basophils). These cells and the neutrophil–lymphocyte ratio (NLR) are crucial for innate and adaptive immune responses to attacks by organisms, and their concentrations are influenced by infection, stress, and inflammation [20,21,22,23]. Elevated WBCs and NLR were associated with increased severity of glycosylated hemoglobin [21]. A prospective observational study in Toronto determined that an increased WBCs and NLR were associated with increased insulin resistance. The researchers concluded that WBCs and NLR can be used to determine the risk of T2DM. As such, both WBCs and NLR are key biological markers for detecting T2DM, and their determination is inexpensive [22]. Higher levels of NLR in patients with T2DM may be linked to the differential influence of hyperglycemia on neutrophils and lymphocytes to underlie the elevated levels of pro-inflammation that underlie insulin resistance [23]. Moreover, it has been reported that high level of WBCs and NLR was significantly positively correlated with active smoking and SHS [24] but negatively correlated with physical activity [25,26]. We, therefore, hypothesize that both smoking status and physical activity level may be associated with T2DM–related inflammation biomarkers, such as WBCs and NLR, which consequently increase susceptibility to T2DM. However, these relationships require clarification.

The purpose of this study was to investigate the effects of SHS and physical inactivity as well as their synergistic effect on the risk of T2DM in Indonesians. Relationships of WBCs and NLR with smoking status, physical activity, and T2DM risk were also estimated.

## 2. Materials and Methods

### 2.1. Research Ethic and Sample Size

The study protocol was reviewed and approved by the Institutional Review Board Ethics Committee No. IRB/009/KET-TPEP/X-2018, the Ethics Committee of Siti Khotidjah Muhammadiyah Sepanjang Hospital and conformed to the provisions of the Declaration of Helsinki. Written informed consent was obtained from each participant after they had received both verbal and written information about the research. The sample size calculation for this study, we estimated sample sizes for both cases and controls by setting the power to (1 − β = 0.8 and the alpha level (α) to 0.01. We posited that the probability of exposure (*p*_0_) among controls would be 0.70 if the true odds ratio (OR; Ψ) for disease in the exposed subjects relative to unexposed subjects was 2.24 [27]. Hence, we required 256 participants with T2DM and 256 control participants. Assuming a 15% dropout rate, the estimated total sample size required 512 participants, so we increased the sample size to 294 for each group, and 588 participants were included.

### 2.2. Study Design and Setting

This study collected primary data using a community-based case–control study with frequency matching by age with a 1:1 ratio. Stratified multistage cluster sampling was used to select participants in East Java, Indonesia, between 1 July–30 November 2018. In the first stage, we selected East Java, a province in Indonesia, for convenience and stratified it into 38 regions. In the second stage cluster, four regions, including urban and rural areas, were selected from the regions. In the third stage, eight community clinics were randomly selected from these four regions for data collection. Three of the clinics refused our invitation to conduct the research. Ultimately, five community clinics joined our study for subject recruitment.

Both the T2DM patients and healthy controls were recruited from five primary clinics in community and were assessed using medical record and brief interview aimed to asses glycemic condition, previous thrombotic events, autoimmune diseases, cancer, chronic and acute diseases. In addition, the clinic medical records of the eligible case and control groups were verified by both physicians and the authors. In total, 588 participants, 294 patients with T2DM and 294 healthy controls, were consecutively recruited. Thus, all participants agreed to participate in the study.

**Figure 1:**
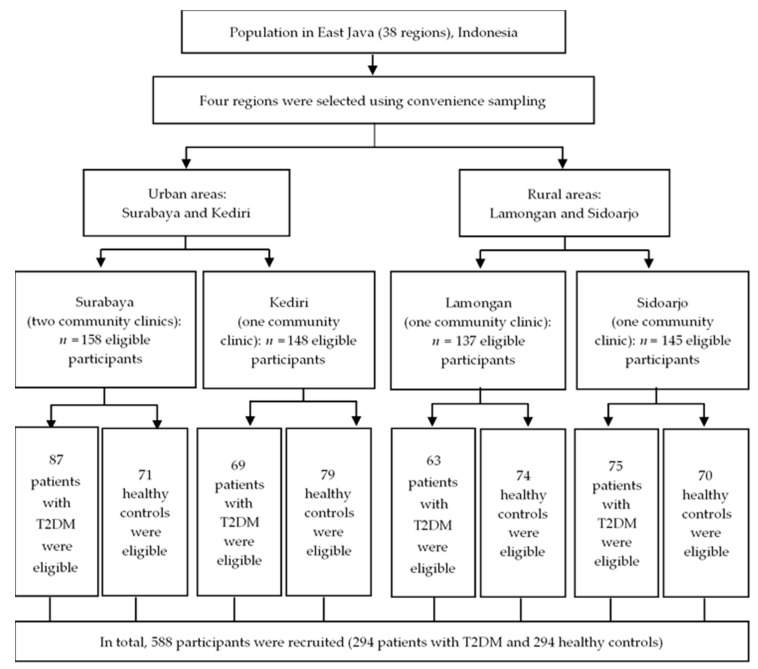
Study sample flowchart.

The study included 294 cases, aged between 18–75 years, with a confirmed diagnosis of type 2 diabetes mellitus by physicians confirmed T2DM was fasting plasma glucose >126 mg/dL (>7.0 mmol/L) based on American Diabetes Association’s criteria [1]. The control group consisted of 294 participants. Eligibility criteria were made corresponding to the, age of controls, and a health status without T2DM-confirmed by physicians. Participants in the control group had fasting plasma glucose 70–99 mg/dL (3.95–5.5 mmol/L) based on the criteria of the American Diabetes Association [1]. Both case and control participants were excluded who (1) had a Mini-Mental State Exam score of ≤24; (2) had any auditory deficiencies; (3) were pregnant; (4) used antidepressants; (5) had an amputated limb or could not walk; (6) had previous thrombotic events, autoimmune diseases, cancer, others chronic and acute diseases. A study sample flowchart is provided in Figure 1.

### 2.3. Instruments and Measures

During the study period, all participants were interviewed by trained nurses and authors using a questionnaire that contained questions about participants’ demographic characteristics, including sex, age, family history of disease, and body mass index. Moreover, clinical and biochemical, and lifestyle behaviors factors including physical activity, smoking status, and food consumption were assessed.

#### 2.3.1. Assessment of Clinical and Biochemical Outcomes

The primary clinical and biological measures included body mass index (BMI), fasting blood glucose (FBG), WBCs, and NLR. Height and weight were measured by research assistants and confirmed using medical records. BMI was calculated as body weight (kg)/height^2^ (m^2^), and we classified participants into two groups: non-obese (BMI < 25 kg/m^2^) and obese (BMI ≥ 25; [28] groups. WBCs, neutrophil, and lymphocyte counts were conducted using an XP-100 automated hematology analyzer (Sysmex, Kobe, Japan) in private laboratory. Both WBCs, and NLR were also classified into two groups using receiver operating characteristic for analysis of odds ratios (ORs) [29]. The cutoff level for WBCs counts was determined to be 7.576 10^3^/µL according to the receiver operating characteristic analysis, and sensitivity and specificity were 0.72 and 0.69. The area under the curve for WBCs was 0.833 (*p* < 0.001). The cutoff level of the NLR was determined to be 1.914 based on the area under the curve; ratio sensitivity and specificity were 0.82 (*p* < 0.001), 0.73, and 0.79, respectively in our study.

#### 2.3.2. Assessment of Physical Activity

Physical activity level was ascertained by estimating MET-h/week for the most recent 12 months [19]. The metabolic equivalent of task (MET) is a unit used to measure caloric expenditure. It is the oxygen expended during a physical activity presented as a ratio to oxygen expended at rest [30]. The intensity of physical activities can be categorized as strenuous, moderate, or mild based on the MET level of the activity [30,31]. MET hours per week (MET-h/week) is used as a record of physical activity [32]. MET-h/week can be categorized into five levels: inactive (<3.75 MET-h/week) and low (3.75–7.49 MET-h/week), moderate (7.50–16.49 MET-h/week), high (16.50–25.49 MET-h/week), and very high (≥25.50 MET-h/week) levels of physical activity [32]. Participants were asked to respond to questions about their regular physical activity during a typical 7-day period (a week), including how many minutes and how many times on average they did each physical activity for more than 15 min, and the intensities of weekly physical activity during a typical week with several examples of activity types given under three intensity categories: strenuous (exhaustion; heart beats rapidly; heart rate of >150 beats/min; e.g., running, soccer, swimming), moderate (not exhausting but some fatigue; heart rate of 120–150 beats/min; e.g., brisk walking, baseball, tennis, easy bicycling), and mild physical activity (minimal effort; heart rate of <120 beats/min; e.g., easy walking, yoga, bowling). The total number of hours and frequency of strenuous, moderate, and mild exercise were multiplied by 9, 5, and 3, respectively. Total weekly physical activities were calculated in arbitrary units by summing the products of the separate components, as in the following formula. For example, if a participant played 30 min of soccer (strenuous) once per week, played 30 min of tennis (moderate) twice a week, and did 30 min of easy walking (mild) five times per week, the weekly total MET-h/week score could be calculated as follows: (9 (strenuous) × 0.5 h/time × 1 time/week) + (5 (moderate) × 0.5 h/time × 2 times/week) + (3 (mild) × 0.5 h/time × 5 times/week) = 4.5 + 5 + 7.5 = 17 MET-h/week.

#### 2.3.3. Assessment of Smoking Status

An active smoker was defined as a person smoking more than one cigarette per day for at least 1 year. A former smoker was defined as a smoker who had quit the smoking habit for 1 year or more. If a participant was neither an active nor former smoker, we sought to evaluate the participant’s exposure to SHS. Subjects were considered SHS if they were exposed to smoke from more than one cigarette per day for at least 1 year. Nonsmokers were participants who were not active, former, or SHS [33].

#### 2.3.4. Assessment by Food Frequency Questionnaire

To determine the usual diets of subjects during the 2 years before T2DM diagnosis, an interviewer-administered short food frequency questionnaire was used, which included main contents of carbohydrates, proteins, fats, fast foods, and fiber as well as a range of the most common Indonesian meals. Participants were asked to self-report their dietary history by indicating the average weekly frequency of consumption of each dietary item as more than once per day, an average of one to six times per week, at least one to three times per month, or never. Nutrient intake was determined using a 25-item Indonesian food composition method [34], which has good performance validity with a content validity index of 0.75 and has been implemented in studies of participants with T2DM in Indonesia [35]. We used means as cutoff values for carbohydrates, protein, fat, fast food, and fiber [34]. This has the advantage of recording categorical data as ordinal, and the inferences made are better able to represent the data and enable a more intuitive interpretation. In this study, they were divided into two levels for each main content with the following cutoff values. The cutoff points were as follow; carbohydrates, <17 and ≥17; protein, <18 and ≥18; fat, <20 and ≥20; fast food, <9 and ≥9; and fiber, ≥2 and <2.

### 2.4. Statistical Analysis

Distributions of demographic characteristics and determinant factors between healthy controls and patients with T2DM were determined using frequency (*n*) and percentages (%) and evaluated using χ^2^ statistics or Fisher’s exact test. Correlations between variables were tested using one-way ANOVA, Pearson’s test, or Spearman’s test, where appropriate. A simple logistic regression was used to calculate the OR and measure the association between risk factors and T2DM in the bivariate analysis. An adjusted OR (AOR) and the corresponding 95% confidence interval (CI) were obtained following the multiple logistic regression for T2DM in relation to exposures of interest (nonsmoker, active smoking, SHS exposure, physical activity, NLR, and WBCs) after adjustment for potential confounding factors in the models (sex, age, family history of T2DM, BMI, and consumption of carbohydrates, protein, fat, fast food, and fiber). Then, the synergistic interaction effect between smoking status with a physical inactivity was investigated after creating six dummy variables for the following six (3 × 2) conditions: (1) nonsmoker and physical activity of ≥7.5 MET-h/weeks (the reference condition or β00); (2) exposed to SHS and physical activity of ≥7.5 MET-h/weeks (β01); (3) active smoker and physical activity of ≥7.5 MET-h/week (β02); (4) nonsmoker and physical activity of <7.5 MET-h/week (β10); (5) exposed to SHS and physical activity of <7.5 MET-h/weeks (β11); and (6) active smoker and physical activity of <7.5 MET-h/weeks (β12). We calculate the additive interaction or synergistic effect using the following categories: (1) if β11 = β01 × β10, and β12 = β02 × β10, no interaction as departure from additivity; (2) if β11 > β01 × β10, and β12 > β02 × β10, positive interaction as departure from additivity; and (3) if β11 < β01 × β10, and β12 < β02 × β10, negative interaction as departure from additivity [36,37]. We also simultaneously assessed the OR and AOR (95% CI) for our synergistic effect using multiple logistic regression models to adjust confounding factors as follows: sex, family history of T2DM, age, BMI, NLR, WBCs, and consumption of carbohydrates, protein, fat, fast food, and fiber. Statistical analyses were performed using SPSS version 25.0 (IBM Corp, Chicago, IL, USA), with a *p* value of <0.05 set as statistically significant.

## 3. Results

The overall demographic characteristics of the participants are summarized in Table 1. Significant differences (*p* < 0.05) were noted in family history of diabetes, BMI, smoking status, physical activity, FBG, NLR, WBCs, and consumption of carbohydrates, protein, fat, fast food, and fiber between patients with T2DM and healthy controls. However, no significant difference in gender or age was revealed between the groups.

Values of the AOR and 95% CI of smoking status, physical activity status, NLR, WBCs, and T2DM risk are presented in Table 2. People exposed to SHS had a 2.69-fold higher risk (95% CI = 1.04–6.99; *p* = 0.042) of having T2DM compared with nonsmokers after adjustment for confounding factors. Participants with physical activity of <7.5 MET-h/week had a 3.90-fold higher risk (95% CI = 1.92–7.90; *p* = 0.001) of having T2DM compared with those with physical activity of ≥7.5 MET-h/week after adjustment for covariate variables. Individuals with a NLR of ≥1.914 had a 4.63-fold higher risk (95% CI = 2.47–8.67; *p* = 0.001) of having T2DM compared with those with a NLR of <1.914 after adjustment for confounders. In addition, participants with a WBCs of ≥7.576 10^3^/µL had a 1.88-fold higher risk (95% CI = 1.05–4.91; *p* = 0.048) of having T2DM compared with those with a WBCs of <7.576 10^3^/µL after adjustment for confounding variables. No significant association was observed between being an active smoker and T2DM after confounding factors were controlled for confounding factors.

The synergistic effect of physical inactivity (<7.5 MET-h/week) and smoking status on T2DM risk is presented in Table 3. A significant synergistic effect of physical inactivity (<7.5 MET-h/week) and both active and SHS on T2DM risk was revealed. The AORs and 95% CIs revealed 7.78-fold (95% CI = 2.39–25.30; *p* = 0.001) and 5.93-fold (95% CI = 1.10–31.91; *p* = 0.038) increases in T2DM risk for SHS with physical activity of <7.5 MET-h/week and active smokers with physical activity of <7.5 MET-h/week, respectively, compared with nonsmokers with a physical activity level of ≥7.5 MET-h/week (Table 3). Furthermore, our study indicated that there was positive synergistic effect (additive interaction) for the combination of SHS and MET-h/week <7.5 on T2DM risk (7.78 > 1.51 × 2.01), also the combination of an active smoker and MET-h/week <7.5 on T2DM risk (5.93 > 1.77 × 2.01).

A significantly different levels (*p* < 0.05) of T2DM–related inflammatory biomarkers such as NLR and WBCs were found in different groups (Table 4). Moreover, a synergistic effect between smoking status (active smoking and SHS) and physical activity of <7.5 MET-h/week to increase the levels of NLR and WBCs were also found (Table 4).

Correlations between smoking status and levels of T2DM–related inflammatory biomarkers such as NLR and WBCs are displayed in Figure S1a,b. NLR was positively correlated (F = 27.31, *p* < 0.001) with smoking status (Figure S1a). In addition, a positive correlation was noted between WBCs and smoking status (F = 24.68, *p* < 0.001; Figure S1b). Moreover, not only NLR (*r* = 0.352, *p* < 0.001) but also WBCs (*r* = 0.402, *p* < 0.001) were positively correlated with an average daily exposure to SHS (Figure S2a,b). Contrarily, both NLR (*r* = −0.394, *p* < 0.001) and WBCs (*r* = −0.297, *p* < 0.001) were negatively correlated with an average physical activity MET-h/week (Figure S3a,b).

## 4. Discussion

To our knowledge, this is the first community-based case–control study to determine that exposure to SHS is significantly correlated with a high NLR and to reveal a synergistic effect of physical activity of <7.5 MET-h/week on increased susceptibility to T2DM. Active smoking and SHS are associated with T2DM risk [6,7]. Oba et al. [38] determined that both active smoking and SHS are associated with impaired glucose tolerance and damage to pancreatic beta cell function, which eventually increases the risk of developing T2DM. In our study, active smokers had an 8.93-fold higher risk of T2DM compared with nonsmokers, but no significant association between active smoking and T2DM was indicated after adjustment for other covariates. This result is similar to that of Idris et al. [9], who recruited 38,052 individuals to estimate potential risk factors of T2DM among Indonesians and found no relationship between active smoking and T2DM. The inconsistent findings might be explained by the low prevalence of active smokers in our study or by other risk factors playing a dominant role over active smoking, thus limiting the power of the analysis.

However, we revealed that exposure to SHS significantly increased the risk of developing T2DM, by 2.69-fold, even after we adjusted for confounding factors. This result is similar to that of a systematic review and meta-analysis, which showed that passive smokers had a 1.22-fold higher risk of having T2DM compared with nonsmokers [6]. SHS for a period of exposure of over 15 years was strongly associated with glucose intolerance and an increased risk of T2DM compared with nonsmokers in the United States [7]. Moreover, one study suggested that SHS, especially for nonsmoking women living with actively smoking husbands, might primarily affect abnormal beta-cells rather than insulin sensitivity in Japanese individuals [38]. SHS increases the level of inhaled cotinine and forced 1-s expiratory volume and enhances blood levels of interleukin-6, a proinflammatory cytokine [39]. Because increased interleukin-6 cytokine is considered to be associated with insulin resistance and beta cell dysfunction, it could be a risk factor for T2DM [40]. Furthermore, the smoke from side-streams has different burning temperatures from mainstream smoke. This can affect the chemical components and their sizes in the smoke [41]. Chemical particles exhaled from side-stream smoke are smaller than those from mainstream smoke [42] and thus may penetrate more deeply into the airways when inhaled as SHS [38]. Such an effect may lead to an increased risk of beta cell dysfunction and insulin resistance, further increasing the risk of T2DM.

Physical inactivity is strongly associated with T2DM risk, whereas walking, occupational activity, and cardiorespiratory fitness were associated with respective 15%, 15%, and 55% decreases in the risk [12]. In the present study, we found that participants with physical activity of <7.5 MET-h/week had a 3.90-fold higher risk of having T2DM. People who engage in light to moderate exercise and those who engage in moderate to vigorous physical activity have −0.12% and −0.25% decreased glycated hemoglobin levels, respectively [13]. In addition, in participants in a study by Boniol et al. [13] who increased their physical activity by 100 min/week, their blood sugar decreased by 4.71 mg/dL. Moreover, moderate to vigorous physical activity is significantly correlated with reduced adiposity, one of the most important T2DM predictors, and decreased levels of related biomarkers, including leptin and interleukin-6 [14,43]. By contrast, an increased level of adiponectin, a protein involved in regulating blood sugar levels and fatty acid breakdown, corresponds with a promotion of physical activity [44]. We suggest advocating increased exercise in people with physical activity of ≥7.5 MET-h/week, which is prominent particularly among Indonesians who are accustomed to inactivity.

In this study, high WBCs and a high NLR were positively associated with T2DM risk. WBCs and NLR are predictors of T2DM. In addition, both the WBCs and NLR count are significantly higher in individuals with T2DM with glycated hemoglobin of >7% than in those with glycated hemoglobin of ≤7%. These data suggest that hyperglycemia may be related to an increased WBCs and NLR [21]. Our study also revealed that T2DM–related inflammatory markers, including WBCs and NLR, are significantly and positively correlated with smoking status, daily average number of cigarettes creating SHS, and physical inactivity. These findings are similar to those of other studies that have determined that both active smoking and SHS increased WBCs, lymphocyte, and granulocyte (neutrophils, eosinophils, and basophils) counts after at least 1 h of active smoking or SHS exposure, and the counts were significantly higher over time [24]. A high neutrophil value is a predictor of a nonspecific inflammatory process that tends to be harmful. However, a low lymphocyte value indicates a relatively inadequate regulation of immune systems [45]. High neutrophil and low lymphocyte levels can increase the NLR [20,45]. In addition, a high NLR is a significant risk factor for insulin resistance among individuals with T2DM [45]. A prospective multi-ethnic cohort study indicated that a high NLR is positively associated with high insulin resistance. The researchers concluded that NLR can be used to determine the risk of T2DM [22]. This mechanism might provide new insight into the pathways that affect how SHS increases NLR, blood glucose, and insulin resistance, which further increases the T2DM risk. In addition, in the present study, physical activity was negatively correlated with WBCs and NLR. Importantly, we found that physical activity of ≥7.5 MET-h/week was significantly correlated with a low NLR among our participants. Our finding is consistent with studies of other diseases in which a higher NLR has been closely associated with lower cardiopulmonary capacity [26]. Therefore, physical activity of ≥3 times/weeks at moderate intensity was inversely associated with NLR among 17,028 asymptomatic adults [46]. In addition, our results are consistent with the findings of Loprinzi et al., [25], who concluded that individuals with moderate to high levels of physical activity were 18.83 times more likely of having reduced WBCs compared with those who had lower activity levels. One mechanism in which physical activity is hypothesized to reduce WBCs and NLR count is through a reduction in inflammation progression. Higher inflammation may play a crucial role in contributing to the progression of T2DM through beta cell damage and insulin resistance [22]. Authors have also suggested that physical activity can reduce adiposity-visceral fat by 10.9% and increase adiponectin levels by 16%, attenuated by central adiposity and loss of fat [14,43,44]. Together, these findings suggest a direct effect of high physical activity on specific inflammation markers, especially neutrophils, lymphocytes, and WBCs. We suggest that individuals who are actively smoke, exposed to SHS, or have activity of <7.5 MET-h/week could have higher levels of inflammatory biomarkers, including WBCs and NLR, and consequently have an increased insulin resistance, which would contribute to the risk of T2DM.

We revealed a synergistic effect between physical activity of <7.5 MET-h/week and active smoking as well as SHS exposure in terms of increased T2DM risk. These results indicate that the Indonesian population must avoid being exposed to both physical activity of <7.5 MET-h/week and SHS as well as active smoke. In addition, regular physical activity of ≥7.5 MET-h/week is a potentially vital factor for protecting active smokers and those exposed to SHS from T2DM risk. The potential mechanism for the synergistic effect between physical activity of <7.5 MET-h/week and SHS as well as active smoke to enhance T2DM risk might be clarified by higher WBCs and NLR. Our findings are in agreement with previous studies that determined a high WBCs and NLR are positively correlated with average daily exposure to SHS [20,24,45] and negatively associated with physical activity of ≥7.5 MET-h/week [25,46]. In addition, high WBCs and NLR are associated with insulin resistance and T2DM risk [22,45]. Therefore, individuals who are exposed to SHS and are also physically inactive can be assumed to have a higher WBCs and NLR as well as a greater risk of T2DM than individuals with only exposure to SHS or who are physical inactivity. We suggest that those with physical activity of ≥7.5 MET-h/week dedicate themselves to reducing inflammatory biomarkers, such as WBCs and NLR count. This would subsequently decrease fasting glucose and glycated hemoglobin levels and contribute to alleviating the risk of T2DM, particularly in individuals who actively smoke or are exposed to SHS [14]. In addition, improving regular physical activity to ≥7.5 MET-h/week and avoiding smoke exposure are potentially valuable strategies for preventing the risk of T2DM.

One of our limitations was that physical activity data included only leisure time activities. Thus, work-related physical activity was not captured by the questionnaire, which may lead an underestimation of the effect of physical inactivity on T2DM. Furthermore, even though our research has adjusted to a large number of possible confounding factors, we cannot exclude the possibility of various types of lymphocytes on NLR. Multiple biomarkers will provide more reliable data to classify those at high risk of prediabetes and subsequent diabetes progression. We only obtained data about WBC and NLR but we didn’t include other biomarkers. Therefore, a future study using more comprehensive biomarkers should be conducted. In addition, a large cluster multisite study on the topic in future study would be beneficial to the generalizability. Due to the case–control study design, we cannot provide evidence of the development of diabetes in our participants. However, we provide a valuable association of SHS and physical inactivity synergistically increased the susceptibility to T2DM. This finding could contribute to identify and promote targeted strategies such as increase physical activity and avoid SHS exposure for preventing the risk of T2DM. These findings indicate that health care practitioners play a key role in identifying and promoting targeted treatment that can prevent the risk of T2DM, such as avoiding SHS exposure and promoting physical activity to ≥7.5 MET-h/week as well as maintaining low NLR levels.

## 5. Conclusions

SHS and physical activity of <7.5 MET-h/week separately and significantly increase the risk of T2DM. A synergistic effect was observed between SHS exposure and physical activity of <7.5 MET-h/week in increasing the risk of T2DM. Both SHS and physical inactivity were significantly correlated with an increase in the levels of T2DM inflammatory biomarkers, namely NLR and WBCs. Our future goal is to develop a protocol of experimental exercises to improve physical activity and ameliorate inflammation biomarkers in Indonesian population to reduce risk T2DM.

## Figures and Tables

**Table 1:** Distributions of demographic characteristics and determinant factors for healthy controls and patients with type 2 diabetes mellitus.

Characteristics	Healthy Controls (*n* = 294) *n* (%)	Patients with T2DM (*n* = 294) *n* (%)	*p* Value *
Sex			0.836
Male	59 (20.1)	57 (19.4)	
Female	235 (79.9)	237 (80.6)	
Family history of diabetes			0.006
No	126 (42.9)	94 (32)	
Yes	168 (57.1)	200 (68)	
Smoking status			<0.001
Nonsmoker	69 (23.5)	15 (5.1)	
SHS exposure	208 (70.7)	246 (83.7)	
Active smoker	17 (5.8)	33 (11.2)	
Age (years), mean ± SD	55.40 ± 6.91	54.44 ± 6.92	1
<55	145 (49.3)	146 (49.7)	0.934
≥55	159 (50.7)	148 (50.3)	
BMI (kg/m^2^), mean ± SD	23.84 ± 3.08	24.46 ± 2.51	0.007
<25	213 (72.4)	178 (60.5)	0.002
≥25	81 (27.6)	116 (39.5)	
Physical activity (MET-h/week within the past one year), mean ± SD	5.79 ± 3.52	2.77 ± 2.36	<0.001
MET ≥7.5	152 (51.7)	21 (7.1)	<0.001
MET <7.5	142 (48.3)	273 (92.9)	
FBG, mean ± SD	80.91± 6.29	304.49 ± 27.04	<0.001
NLR, mean ± SD	1.72 ± 0.23	2.12 ± 0.42	0.023
<1.914	234 (79.6)	79 (26.9)	<0.001
≥1.914	60 (20.4)	215 (73.1)	
WBCs (10^3^/µL), mean ± SD	6.96 ± 0.84	8.20 ± 1.02	<0.001
<7.576	204 (69.4)	84 (28.6)	<0.001
≥7.576	90 (30.6)	210 (71.4)	
Carbohydrate consumption score, mean ± SD	18.33 ± 2.76	19.90 ± 2.23	<0.001
<19.12	175 (59.5)	69 (23.5)	<0.001
≥19.12	119 (40.5)	225 (76.5)	
Protein consumption score, mean ± SD	18.44 ± 2.15	18.44 ± 1.68	<0.001
<18.44	169 (57.5)	34 (11.6)	<0.001
≥18.44	125 (42.5)	260 (88.4)	
Fat consumption score, mean ± SD	18.17 ± 2.74	21.13 ± 2.92	<0.001
<19.65	214 (72.8)	86 (29.3)	<0.001
≥19.65	80 (27.2)	208 (70.7)	
Fast food consumption score, mean ± SD	7.60 ±2.01	9.30 ± 2.53	<0.001
<8.45	201 (68.4)	115 (39.1)	<0.001
≥8.45	93 (31.6)	179 (60.9)	
Fiber consumption score, mean ± SD	2.85 ± 1.23	2.39 ± 0.80	<0.001
≥2.62	274 (93.2)	137 (46.6)	<0.001
<2.62	20 (6.8)	157 (53.4)	

Note: T2DM: type 2 diabetes mellitus; SD: standard deviation; SHS: secondhand smoke; BMI: body mass index; MET: metabolic equivalent of task; FGB: fasting blood glucose; NLR: neutrophil–lymphocyte ratio; WBCs: white blood cells. * Data were presented as mean ± SD, frequency and percentage, and *p*-values were calculated using independent sample *t*-test, Chi-square test, or Fisher’s exact test, where appropriate. A *p* value of <0.05 indicates statistical significance.

**Table 2:** Adjusted odds ratios and 95% confidence intervals for smoking status, physical inactivity, diabetes mellitus–associated inflammatory biomarkers, and type 2 diabetes mellitus risk (*n* = 588).

Variables	Healthy Controls (*n* = 294) *n* (%)	Patients with T2DM (*n* = 294) *n* (%)	Unadjusted OR (95% CI)	AOR (95% CI)
Smoking status				
Nonsmoker	69 (23.5)	15 (5.1)	1.00	1.00
SHS exposure	208 (70.7)	246 (83.7)	5.44 (3.02–9.79) **	2.69 (1.04–6.99) *
Active smoker	17 (5.8)	33 (11.2)	8.93 (3.97–20.04) **	2.03 (0.61–8.62)
MET-h/week within the past one year				
MET-h/week ≥7.5	152 (51.7)	21 (7.1)	1.00	1.00
MET-h/week <7.5	142 (48.3)	273 (92.9)	13.92 (8.45–22.93) **	3.90 (1.92–7.90) **
NLR				
<1.914	234 (79.6)	79 (26.9)	1.00	1.00
≥1.914	60 (20.4)	215 (73.1)	10.61 (7.23–15.57) **	4.63 (2.47–8.67) **
WBCs (10^3^/µL)				
<7.576	204 (69.4)	84 (28.6)	1.00	1.00
≥7.576	90 (30.6)	210 (71.4)	5.67 (3.97–8.07) **	1.88 (1.05–4.91) *

Note: T2DM: type 2 diabetes mellitus; OR: odds ratio; CI: confidence interval; AOR: adjusted odds ratio; SHS: secondhand smoke; MET: metabolic equivalent of task; NLR: neutrophil–lymphocyte ratio; WBCs: white blood cells. AOR, adjusted for gender, age, family history of diabetes, body mass index, and scores for the consumption of carbohydrates, protein, fat, fast food, and fiber. * *p* value of < 0.05, ** *p* value of < 0.001.

**Table 3:** Adjusted odds ratio and 95% confidence interval for the synergistic effect of smoking status and physical activity on type 2 diabetes mellitus risk (*N* = 588).

Variables	Healthy Controls (*n* = 294) *n* (%)	Patients with T2DM (*n* = 294) *n* (%)	Unadjusted OR (95% CI)	AOR (95%CI)
Nonsmoker and MET-h/week ≥7.5	49 (16.7)	7 (2.4)	1.00	1.00
Exposed to SHS and MET-h/week ≥7.5	93 (31.6)	8 (2.7)	0.60 (0.20–1.75)	1.51 (0.38–5.95)
Active smoker and MET-h/week ≥7.5	10 (3.4)	6 (2.0)	4.20 (1.16–15.18) *	1.77 (0.27–11.47)
Nonsmoker and MET-h/week <7.5	20 (6.8)	8 (2.7)	2.80 (0.89–8.75)	2.01 (0.46–8.88)
Exposed to SHS and MET-h/week <7.5	115 (39.1)	238 (81.0)	14.49 (6.36–32.97) ***	7.78 (2.39–25.30) **
Active smoker and MET-h/week <7.5	7 (2.4)	27 (9.2)	27.00 (8.56–85.11) ***	5.93 (1.10–31.91) *

Note: T2DM: type 2 diabetes mellitus; OR: odds ratio; CI: confidence interval; AOR: adjusted odds ratio; SHS: secondhand smoke; MET: metabolic equivalent of task. AOR, adjusted for gender, age, family history of diabetes, body mass index, neutrophil–lymphocyte ratio; white blood cells and scores for the consumption of carbohydrates, protein, fat, fast food, and fiber. * *p* value of < 0.05, ** *p* value of < 0.01, *** *p* value of < 0.001.

**Table 4:** Association between the levels of inflammatory biomarkers and the status of smoking and physical activity (*n* = 588).

Variables	Nonsmoker and MET-h/Week ≥7.5	Exposed to SHS and MET-h/Week ≥7.5	Active Smoker and MET-h/Week ≥7.5	Nonsmoker and MET-h/Week <7.5	Exposed to SHS and MET-h/Week <7.5	Active Smoker and MET-h/Week <7.5	*p* Value
NLR (mean ± SD)	1.61 ± 0.17	1.65 ± 0.21	2.16 ± 0.63	1.86 ± 0.27	2.01 ± 0.38	2.19 ± 0.39	<0.001 ^a^
NLR Mean difference (95% CI) ^b^	Ref	0.03 (−0.16–0.22)	0.67 (0.29–1.05) **	0.24 (−0.03–0.51)	0.39 (0.22–0.55) **	0.53 (0.29–0.77) **	
WBCs (10^3^/µL) (mean ± SD)	6.73 ± 0.68	7.01 ± 0.91	7.87 ± 1.41	7.17 ± 0.84	7.83 ± 1.12	8.22 ± 1.12	<0.001 ^a^
WBCs (10^3^/µL) Mean difference (95% CI) ^b^	Ref	0.28 (−0.29–0.86)	1.48 (0.34–2.62) *	0.44 (−0.36–1.240)	1.10 (0.63–1.60) **	1.34 (0.62–2.06) **	

Abbreviation: SD: standard deviation; SHS: secondhand smoke; MET: metabolic equivalent of task; NLR: neutrophil–lymphocyte ratio; WBCs: white blood cells. ^a^ One-way ANOVA, ^b^ Scheffé post hoc analysis; * *p* value >0.05; ** *p* value > 0.001.

## Supplementary Materials

The following are available online at https://www.mdpi.com/1660-4601/17/16/5696/s1, Figure S1: correlation between smoking status (nonsmokers, those exposed to secondhand smoke, or active smokers) and (a) neutrophil–lymphocyte ratio (NLR); (b) white blood cell count (WBC), an inflammatory-biomarkers (*n* = 588), Figure S2: correlation between average daily exposure to secondhand smoke (number of cigarettes) and (a) neutrophil–lymphocyte ratio; (b) white blood cell count (WBC), an inflammatory-biomarkers *(n* = 538), Figure S3: correlation between physical activity (metabolic equivalent of task (MET)-h/week in previous month) and (a) neutrophil–lymphocyte ratio; (b) white blood cell count (WBC), an inflammatory-biomarkers *(n* = 538).
