# Supplementary material for: Secondhand Smoke Correlates with Elevated Neutrophil-Lymphocyte Ratio and Has a Synergistic Effect with Physical Inactivity on Increasing Susceptibility to Type 2 Diabetes Mellitus: A Community-Based Case Control Study
Source: Int J Environ Res Public Health. 2020 Aug 6;17(16):5696. doi: 10.3390/ijerph17165696 (PMC7459643; doi:10.3390/ijerph17165696)
Supplement: Supplementary file 1 [file ijerph-17-05696-s001.pdf]

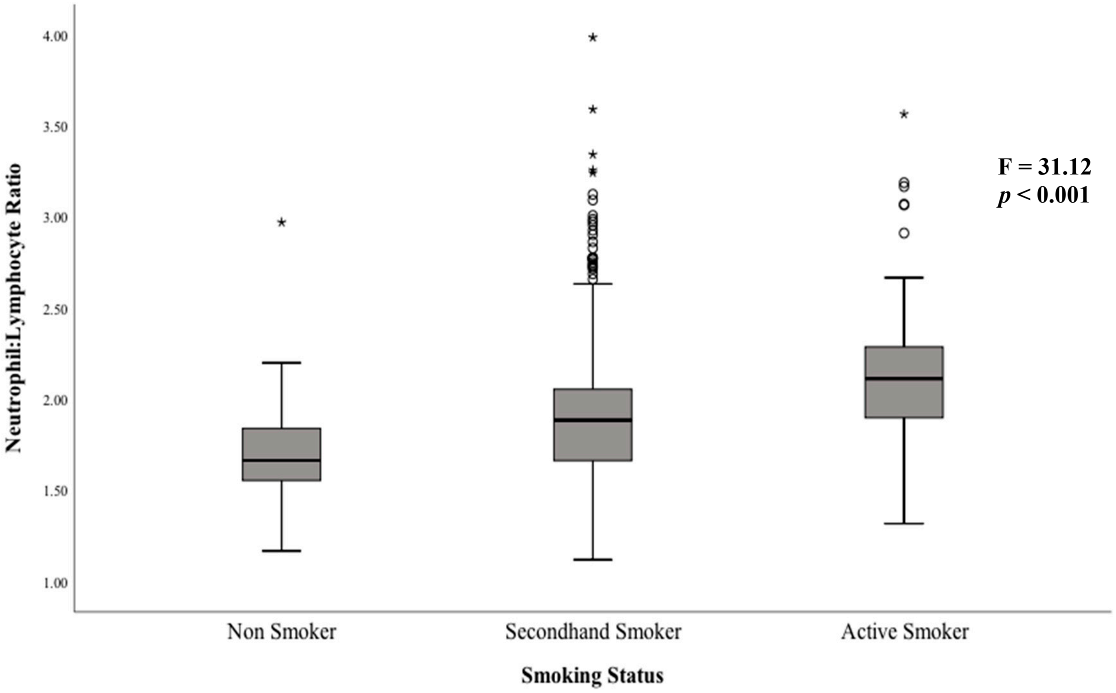

(a)

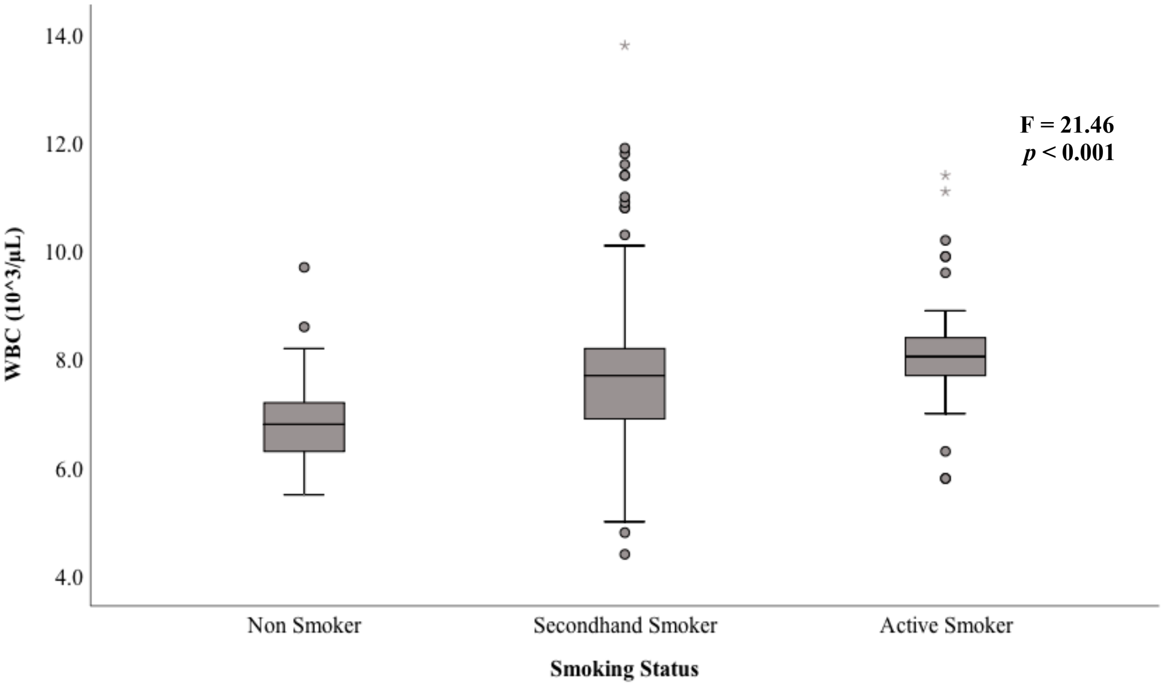

(b)

Figure 1. Correlation between smoking status (nonsmokers, those exposed to secondhand smoke, or active smokers) and (a) neutrophil–lymphocyte ratio (NLR); (b) white blood cell count (WBC), an inflammatory-biomarkers (n = 588).

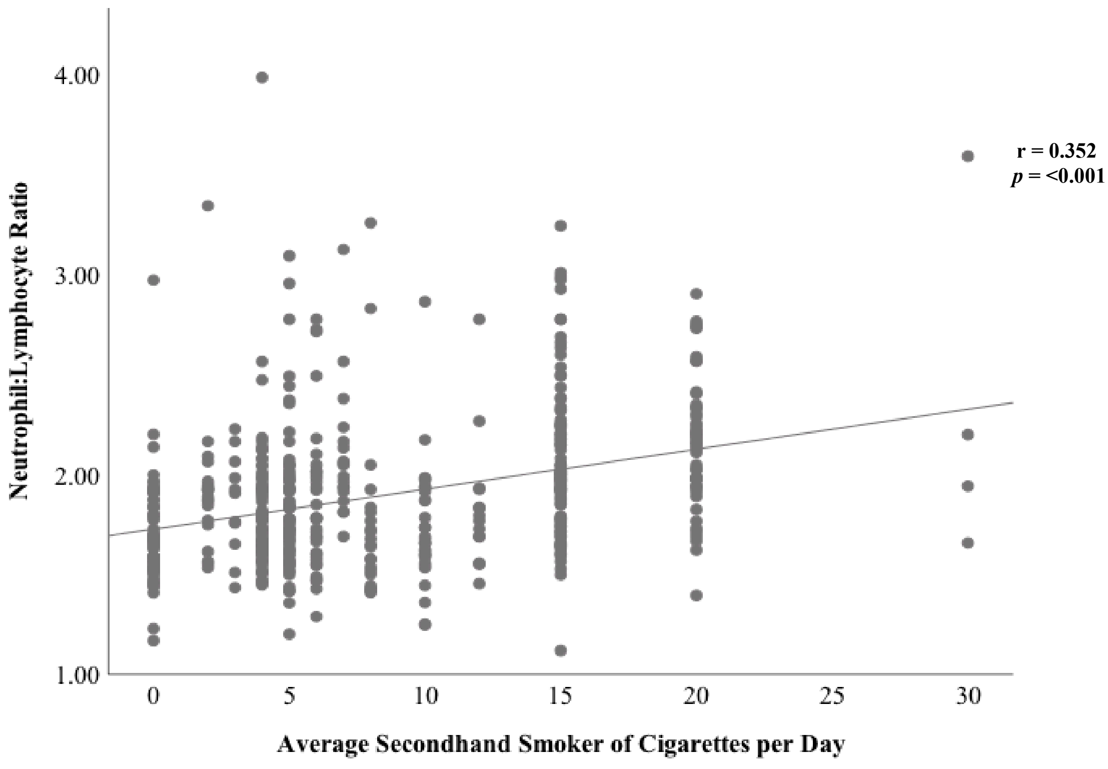

(a)

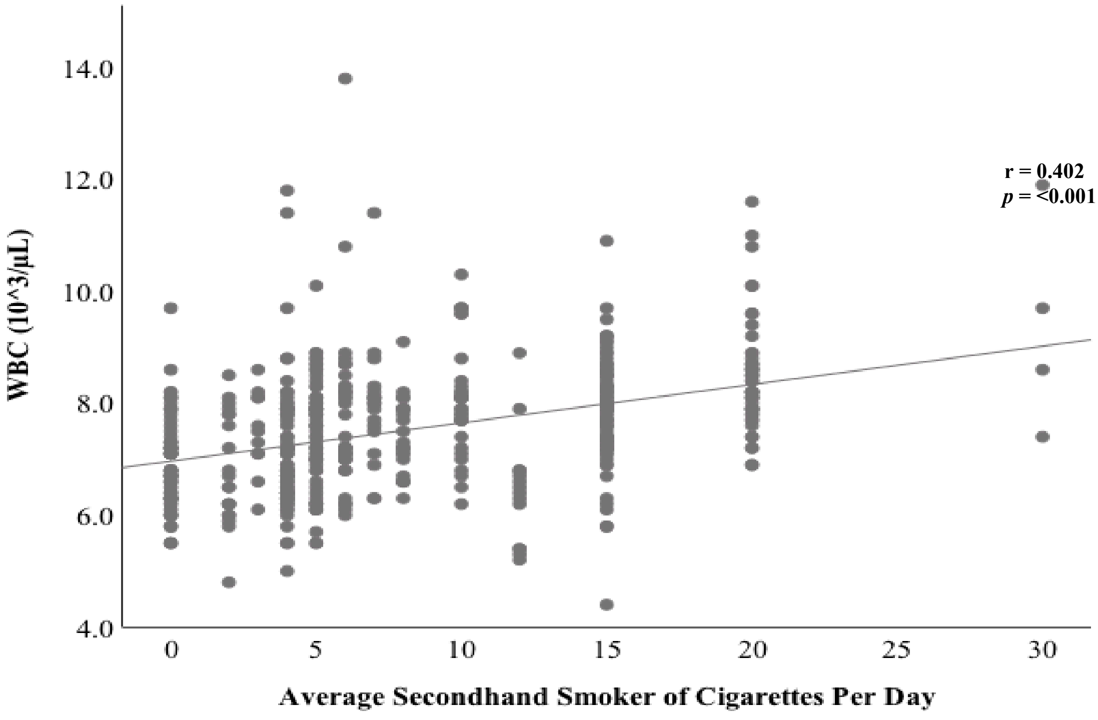

(b)

Figure S2. Correlation between average daily exposure to secondhand smoke (number of cigarettes) and (a) neutrophil–lymphocyte ratio; (b) white blood cell count (WBC), an inflammatory-biomarkers (n = 538).

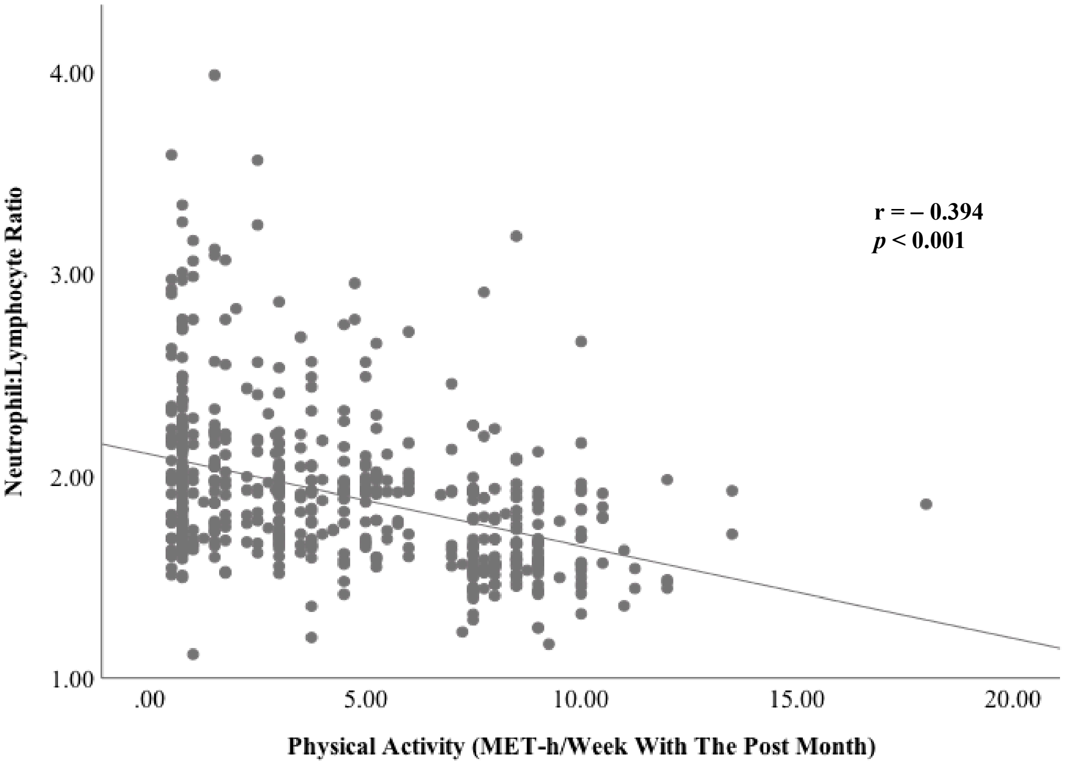

(a)

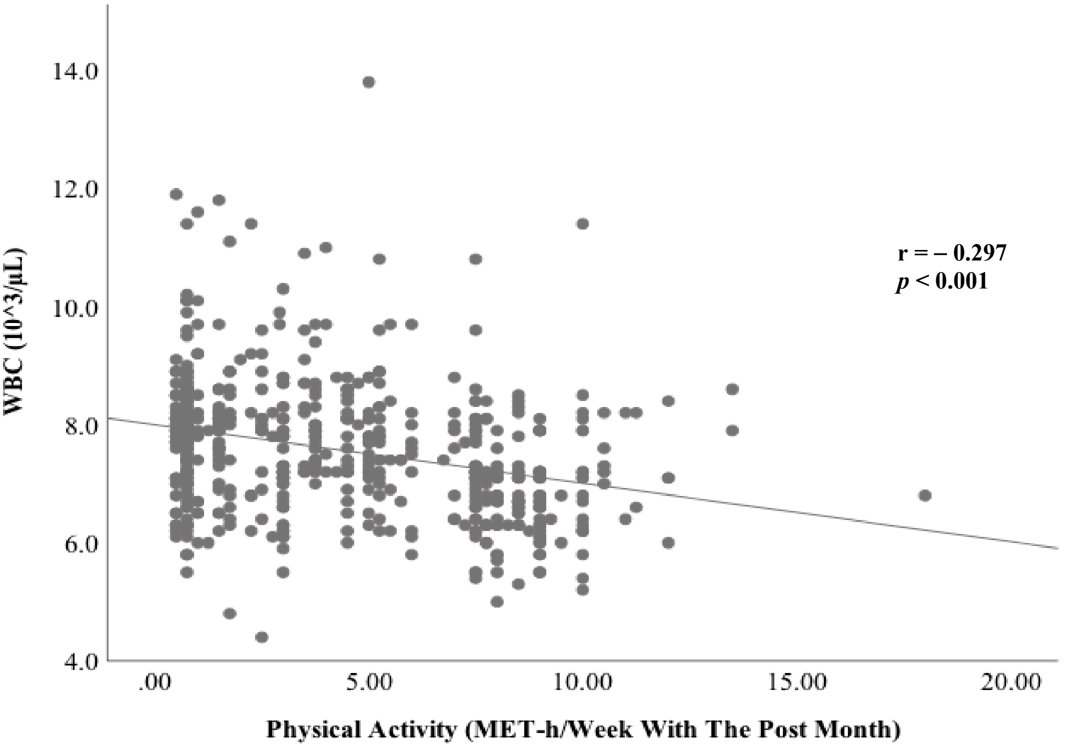

(b)

Figure S3. Correlation between physical activity (metabolic equivalent of task [MET]-h/week in previous month) and (a) neutrophil–lymphocyte ratio; (b) white blood cell count (WBC), an inflammatory-biomarkers (*n* = 538).
